# Climate factors dominate the elevational variation in grassland plant resource utilization strategies

**DOI:** 10.3389/fpls.2024.1430027

**Published:** 2024-08-07

**Authors:** Jinkun Ye, Yuhui Ji, Jinfeng Wang, Xiaodong Ma, Jie Gao

**Affiliations:** ^1^ Key Laboratory for the Conservation and Regulation Biology of Species in Special Environments, College of Life Science, Xinjiang Normal University, Urumqi, China; ^2^ Key Laboratory of Earth Surface Processes of Ministry of Education, College of Urban and Environmental Sciences, Peking University, Beijing, China

**Keywords:** climatic factors, elevation, key leaf traits, resource utilization strategies, soil nutrients

## Abstract

Specific leaf area (SLA) and leaf dry matter content (LDMC) are key leaf functional traits often used to reflect plant resource utilization strategies and predict plant responses to environmental changes. In general, grassland plants at different elevations exhibit varying survival strategies. However, it remains unclear how grassland plants adapt to changes in elevation and their driving factors. To address this issue, we utilized SLA and LDMC data of grassland plants from 223 study sites at different elevations in China, along with climate and soil data, to investigate variations in resource utilization strategies of grassland plants along different elevational gradients and their dominant influencing factors employing linear mixed-effects models, variance partitioning method, piecewise Structural Equation Modeling, etc. The results show that with increasing elevation, SLA significantly decreases, and LDMC significantly increases (*P* < 0.001). This indicates different resource utilization strategies of grassland plants across elevation gradients, transitioning from a “faster investment-return” at lower elevations to a “slower investment-return” at higher elevations. Across different elevation gradients, climatic factors are the main factors affecting grassland plant resource utilization strategies, with soil nutrient factors also playing a non-negligible coordinating role. Among these, mean annual precipitation and hottest month mean temperature are key climatic factors influencing SLA of grassland plants, explaining 28.94% and 23.88% of SLA variation, respectively. The key factors affecting LDMC of grassland plants are mainly hottest month mean temperature and soil phosphorus content, with relative importance of 24.24% and 20.27%, respectively. Additionally, the direct effect of elevation on grassland plant resource utilization strategies is greater than its indirect effect (through influencing climatic and soil nutrient factors). These findings emphasize the substantive impact of elevation on grassland plant resource utilization strategies and have important ecological value for grassland management and protection under global change.

## Introduction

Plant functional traits are closely related to plant survival, growth, and reproduction. It not only can reflect plant responses to environmental changes and adaptation strategies, but also influencing the functions of individual plants and ecosystems (Song et al., 2024). For example, specific leaf area (SLA) and leaf dry matter content (LDMC) are commonly used to represent plant resource utilization strategies (Kattge et al., 2020; Liu et al., 2021). Among them, specific leaf area is the ratio of leaf area to dry mass (Worthy et al., 2020), reflecting a plant’s light resource utilization and allocation strategies (Liu et al., 2023), and leaf dry matter content is the ratio of leaf dry mass to fresh mass, indicating a plant’s nutrient conservation capacity (Smart et al., 2017). Typically, in favorable environments, plants exhibit higher SLA and lower LDMC, displaying a “faster investment-return” resource utilization strategy (Májeková et al., 2014). However, in harsh environments such as those with nutrient and water scarcity, plants tend to reduce SLA and increase LDMC in order to better withstand adverse conditions and prolong leaf lifespan, exhibiting a “slower investment-return” resource utilization strategy (Dwyer et al., 2014; Gong and Gao, 2019).

Grasslands cover approximately one quarter of the global terrestrial area and are an important component of terrestrial ecosystems (Villoslada Peciña et al., 2019). Grasslands also have a very wide distribution, ranging from low to high elevations (New, 2019). The vertical range of grassland distribution in our country is also very wide (Yu et al., 2021; Shen et al., 2022). Existing studies have shown differences in adaptation strategies among grassland plants at different elevations, but there is currently no comprehensive research on the adaptation strategies of plants at different elevations in China. To fill this gap in China, we obtained data from published authoritative papers covering 223 study sites at different elevations in China (i.e., SLA, LDMC) with the aim of exploring the adaptive strategies of grass plants at different elevations in China. Furthermore, exploring the resource utilization strategies of grassland plants at different elevational gradients and their controlling factors has significant ecological value for understanding the specific impacts of global change on grassland plants, and provides theoretical basis and important guiding significance for grassland management and conservation under global change (Fontana et al., 2017).

Different elevations and environments result in different adaptation strategies for plants (Liu et al., 2021). For instance, in high-elevation areas, due to low temperatures, strong ultraviolet radiation, and short growing seasons, herbaceous plants must maximize the use of the environment through traits adapted to habitat conditions (Abbas et al., 2022). Moreover, Plants living at high elevational gradients exhibit cold-tolerant traits; they generally grow slowly but have long lifespans, are small in size, and have thick leaves (Hultine and Marshall, 2000), with high LDMC and low SLA, adopting a “slower investment-return” resource utilization strategy to achieve survival and reproduction. However, plants living at middle and lower elevations generally have higher SLA and lower LDMC to quickly acquire ample resources, exhibiting a “faster investment-return” resource utilization strategy (Islam et al., 2024). In general, SLA decreases with increasing elevation, while LDMC increases with elevation, indicating that plants choose different resource utilization strategies at different elevational gradients (Rixen et al., 2022). However, most current studies on the elevation variation of grassland plant resource strategies have been conducted at small, localized scales (Fontana et al., 2017; Wieczynski et al., 2019; Kramp et al., 2022; Feng et al., 2023), lacking integration and synthesis of these findings at broader macroscopic scales. It remains unknown whether grassland plant resource utilization strategies and their driving factors at large scales are similar to those observed at local scales. Therefore, there is an urgent need for research on the elevation variation of grassland plant resource utilization strategies and their driving factors at larger scales, aiming to provide valuable insights for guiding grassland management and conservation under global climate change scenarios.

Numerous studies have found that plant resource utilization strategies are influenced by various ecological factors (Huang et al., 2021; Wang et al., 2022a). Similarly, plants adapt to different environments by adjusting the SLA and LDMC of their leaves. For example, as precipitation and temperature decrease, plants adopt a conservative approach to resist moisture stress and cold stress, with a significant reduction in SLA and an increase in LDMC. This leads to slower growth and greater investment in leaf construction, adopting a “slower investment-return” resource utilization strategy (Firn et al., 2019). However, plants exhibited higher SLA and lower LDMC when soil moisture increased and soil temperatures rose because soil nutrient availability was higher at this time, and plants needed to increase the plant’s net photosynthetic rate and transpiration rate, displaying a “faster investment-return” resource utilization strategy (Pietsch et al., 2014; Gong and Gao, 2019). In addition to water and thermal conditions, the duration of sunlight also significantly affects plant resource utilization strategies. For instance, in low light conditions, to capture more light, plants increase leaf area and reduce leaf thickness to enhance SLA, adopting a “faster investment-return” resource utilization strategy (Lusk et al., 2008).

Among many environmental factors, soil, as the direct living environment of plants, soil physical and chemical properties and soil nutrient factors have significantly impact on plant resource utilization strategies (Wang et al., 2021; Cui et al., 2022). For example, plants in acidic soils have higher SLA and lower LDMC, enabling more efficient nutrient uptake (Tao et al., 2019). Plant species living in arid and barren environments tend to conserve more nutrients in plant tissues that are long-lived and resistant, thus exhibiting a “slower investment-return” resource utilization strategy (Kramp et al., 2022). Furthermore, soil nutrients, particularly nitrogen (N) and phosphorus (P), significantly affect plant photosynthesis (Yang et al., 2017), e.g., as soil nitrogen content increases, SLA tends to increase and LDMC tends to decrease (Hodgson et al., 2011), leading plants to adopt a “faster investment-return” resource utilization strategy. Therefore, to explore the effects of environmental factors on plant adaptive strategies, we also analyzed the relationship between different environmental factors and SLA and LDMC.

Based on data from 223 field grassland sites at various elevations in China, this study aims to explore the differences in resource utilization strategies of grassland plants across different elevation gradients and their dominant factors. To address the above issues, we propose the following hypotheses: (1) As the elevation gradient increases, the resource utilization strategy of grassland plants shifts from “fast investment-return” to “slower investment-return”; (2) Climatic factors are the dominant environmental factors influencing the resource utilization strategies of grassland plants at different elevational gradients, with soil nutrient factors also playing a significant coordinating role; (3) The direct effect of elevation on the resource utilization strategy of grassland plants is greater than its indirect effects.

## Materials and methods

### SLA and LDMC data

China’s vast territory and diverse climates make it a major grassland country in the world. Furthermore, the elevational range of grasslands in China is broad (Yu et al., 2022; Shen et al., 2022). The continuous variation in elevation provides conditions for exploring the resource utilization strategies of grassland plants at different elevational gradients (Goll et al., 2017).

**Figure 1:**
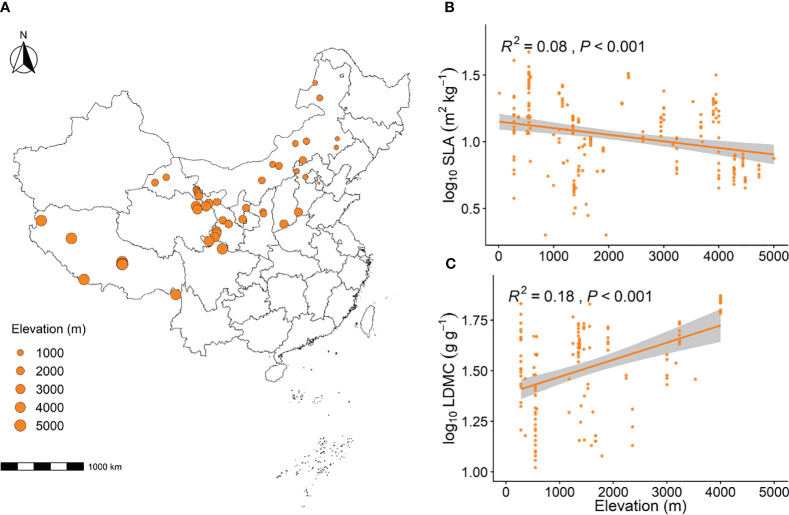
Geographic distribution of grassland sampling sites **(A)** and the linear relationship between elevation and SLA **(B)** and LDMC **(C)**. The size of the circles at the sampling sites represents the relative elevation. SLA stands for specific leaf area, and LDMC represents leaf dry matter content. Both SLA and LDMC data have been log-transformed. *R* ^2^ indicates the model’s goodness of fit, and the *P*-value indicates the level of significance. The shaded area shows a 95% confidence interval.

In this study, we conducted a literature search on two major websites, namely the China National Knowledge Infrastructure (CNKI) and Web of Science, using “China”, “grassland”, “LDMC”, and “SLA” as keywords. We collected data from some of these sources, covering studies from 2004 to 2022. The time span of this period is small enough to minimize the impact of time on this study. We collected data from 223 grassland sites. These grassland sites ranged from 27.8°N to 50.2°N latitude and 79.72°E to 121.1°E longitude, encompassing temperate continental, temperate monsoon, and alpine mountain climates. The elevation ranges from 13 meters to 5,000 meters (**Figure 1A**).

In the process of literature data selection, we followed several principles: (1) Studies that specifically address SLA and LDMC at the community level within Chinese grassland ecosystems, far from human interference, are necessary to ensure the data is directly applicable to the research question; (2) Prioritize peer-reviewed articles and studies conducted by reputable institutions to ensure the reliability and accuracy of the data; (3) Studies that measure SLA and LDMC using similar study designs and methods, such as sampling time, sampling methods, laboratory measurement, and analytical techniques, should strive for consistency to ensure comparability of data across different studies; (4) Studies from diverse geographical locations within China to cover a broad range of elevations and grassland types, ensuring a comprehensive understanding of the variations; (5) Prioritize studies with large sample sizes and appropriate plot sizes to enhance the reliability of the results; (6) The selected literature should provide as complete, detailed, and standardized data as possible, including necessary geographic information such as latitude, longitude, and elevation, as well as statistical information such as means, standard deviations, or standard errors of the data, which ensures comparability and integration for rigorous analysis and accurate results; (7) The selected literature should have the smallest possible time span to minimize the impact of time on this study.

The specific experimental methods and calculation methods for SLA and LDMC are as follows:

At each sampling site, randomly select at least four plots of vegetation typical to the region, each larger than 10 m × 10 m. Within each plot, establish at least three quadrats larger than 1 m × 1 m. Collect leaves from each herbaceous plant in the north, south, east, and west directions, mix them, and place them in moisture-preserving bags for transport to the laboratory for SLA and LDMC measurements. Measure the fresh leaf area using a leaf area meter after removing the petioles, and measure the fresh mass of the leaves using an electronic balance with an accuracy of 0.1 mg. Subsequently, place the fresh leaves in an oven at approximately 105°C for high-temperature blanching, and then dry them at 60-70°C for 48-72 hours. Weigh the dried leaves using an electronic balance with an accuracy of 0.1 mg to obtain the dry mass. Calculate SLA as the ratio of leaf area to leaf dry mass, and calculate LDMC as the ratio of leaf dry mass to leaf fresh mass. Calculate the community-weighted mean (CWM) of grassland SLA and LDMC based on species abundance. Due to the varying units used for SLA and LDMC across different studies, convert the units uniformly to m²/kg for SLA and g/g for LDMC before data analysis.

### Environmental data

The environmental data used in this study includes annual mean temperature (MAT), coldest month mean temperature (MACT), hottest month mean temperature (MAHT), and mean annual precipitation (MAP) were extracted from the WorldClim global climate database (https://www.worldclim.org/, accessed on 1 July 2023) at a spatial resolution of 1 km. Annual sunshine duration (ASD) and mean annual evaporation (MAE) were both extracted from the Meteorological Data Center of the China Meteorological Administration (http://data.cma.cn/site/index.html, accessed on 1 July 2023) at a spatial resolution of 1 km. Soil pH, soil nitrogen, and available soil phosphorus within the top 30 cm of the soil layer were extracted from a 250 m resolution grid (http://www.csdn.store, accessed on 1 July 2023; https://www.osgeo.cn/data/wc137, accessed on 1 July 2023).

### Data analysis

SLA and LDMC data were log_10_-transformed before analyses to improve data distributions. All statistical analyses were conducted in R (version 4.3.1, R Core Team, 2023). To simultaneously account for fixed effects such as elevation, climate, and soil factors influencing SLA and LDMC, as well as random effects arising from different sampling sites and plant species, in order to enhance the precision and reliability of data analysis, we used linear mixed-effects models to explore the impact of elevation on grassland plant resource utilization strategies (characterizing with SLA and LDMC) and analyze differences in resource utilization strategies among grassland plants at different elevational gradients. Additionally, linear mixed-effects models also are employed to analyze changes in climatic and soil factors along elevational gradients and their effects on SLA and LDMC, thereby assessing the impact of climatic and soil factors on resource utilization strategies of grassland plants at different elevations. This analysis was conducted using the “lme4” package in R. *R*-squared represents the model’s goodness of fit, and the *P*-value indicates significance.

Climatic factors primarily include mean annual temperature (MAT), coldest month mean temperature (MACT), hottest month mean temperature (MAHT), mean annual precipitation (MAP), mean annual evaporation (MAE), and annual sunshine duration (ASD). Soil factors mainly include soil nitrogen content (Soil N), soil phosphorus content (Soil P), and soil pH. Considering potential multicollinearity among these factors, we use the “linkET” package in R to perform multivariate correlation analysis to elucidate the interrelationships among the influencing factors.

We employed variance partitioning methods to quantify the explanatory power of climatic and soil factors on the spatial variation of SLA and LDMC at different elevational gradients. The variance decomposition analysis was completed using the “rdacca.hp” package in R. A machine learning approach using boosted regression trees was utilized, with significance testing conducted at the 0.05 level, to explore the independent contributions of each potential influencing factor to the spatial variability of grassland plant resource utilization strategies. This analysis was performed using the “gbm” package in R.

We used piecewise Structural Equation Modeling (piecewiseSEM) to explore the pathways through which each influencing factor within the climatic and soil variables affects the SLA and LDMC of grassland plants. To assess the robustness of the relationships between key ecological factors and SLA and LDMC, we utilized piecewiseSEM to account for the random effects of sampling sites and to provide “marginal” and “conditional” contributions of environmental predictors. These analyses were implemented in R using the “piecewiseSEM”, “nlme”, and “lme4” packages. The goodness of fit for the models was assessed using Fisher’s exact test. Based on acceptable model criteria, specifically a significance level of *P* < 0.05 and optimal model fit (0 ≤ Fisher’s C/*df* ≤ 2 and 0.05 < *P* ≤ 1.00), the models were progressively refined and enhanced to select the best model.

## Results

### Elevational patterns in investment strategies of grassland plants

With the increase in elevation gradient, grassland SLA significantly decreased (*P* < 0.001, **Figure 1B**), while grassland LDMC significantly increased (*P* < 0.001, **Figure 1C**). As the elevation gradient increased, the MAT, the MAHT, and the MAE all significantly decreased, whereas the MAP and the ASD both significantly increased (*P* < 0.001, **Supplementary Figure S1**). With the rise in elevation, the soil N and soil P significantly increased (*P* < 0.01, **Supplementary Figure S2**).

### Climatic factors influencing investment strategies of grassland plants

**Figure 2:**
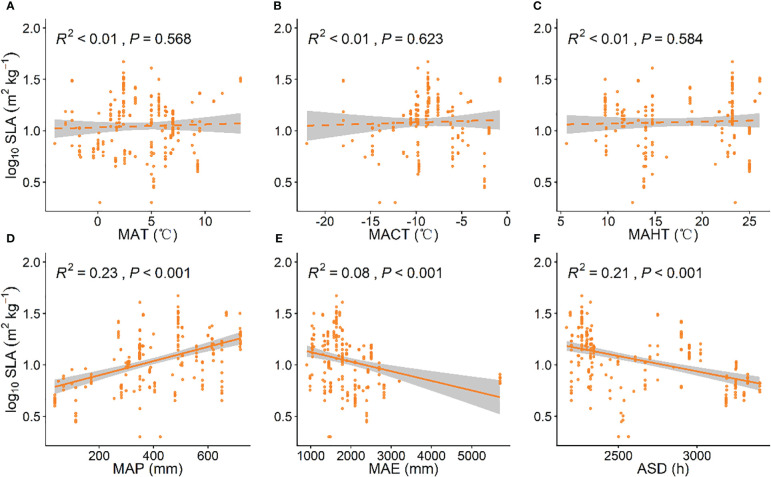
Linear relationships between climatic factors and specific leaf area (SLA). **(A)** mean annual temperature (MAT); **(B)** coldest month mean temperature (MACT); **(C)** hottest month mean temperature (MAHT); **(D)** mean annual precipitation (MAP); **(E)** mean annual evaporation (MAE); **(F)** annual sunshine duration (ASD). SLA data have been log-transformed. *R* ^2^ indicates the model’s goodness of fit, and the *P*-value indicates the level of significance. The shaded area shows a 95% confidence interval.

**Figure 3:**
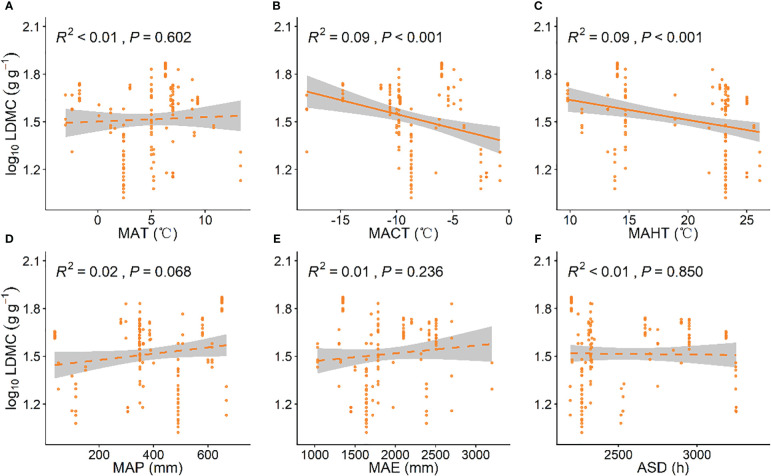
Linear relationships between climatic factors and leaf dry matter content (LDMC). **(A)** mean annual temperature (MAT); **(B)** coldest month mean temperature (MACT); **(C)** hottest month mean temperature (MAHT); **(D)** mean annual precipitation (MAP); **(E)** mean annual evaporation (MAE); **(F)** annual sunshine duration (ASD). LDMC data have been log-transformed. *R* ^2^ indicates the model’s goodness of fit, and the *P*-value indicates the level of significance. The shaded area shows a 95% confidence interval.

As MAP increased, grassland plant SLA significantly increased (*P* < 0.001, **Figure 2D**), while with increases in ASD and MAE, grassland plant SLA significantly decreased (*P* < 0.001, **Figures 2E, F**). Grassland LDMC showed a significant negative correlation with the MACT and MAHT (*P* < 0.001, **Figures 3B, C**). As MACT and MAHT increased, grassland LDMC significantly decreased (**Figures A–C**). With rising temperatures and increasing precipitation, grassland plants shifted to a “faster investment-return” survival strategy. Among all climatic factors, MAP and ASD had better predictive power for grassland plant SLA (*R* ^2 = ^0.23, *P* < 0.001; **Figure 2D**; *R* ^2^ = 0.21, *P* < 0.001; **Figure 2F**), while MACT and MAHT had similar predictive effects on grassland LDMC (*R* ^2^ = 0.09, *P* < 0.001, **Figure 3B**; *R* ^2^ = 0.09, *P* < 0.001, **Figures 3A–F**).

### Soil nutrient factors influencing investment strategies of grassland plants

**Figure 4:**
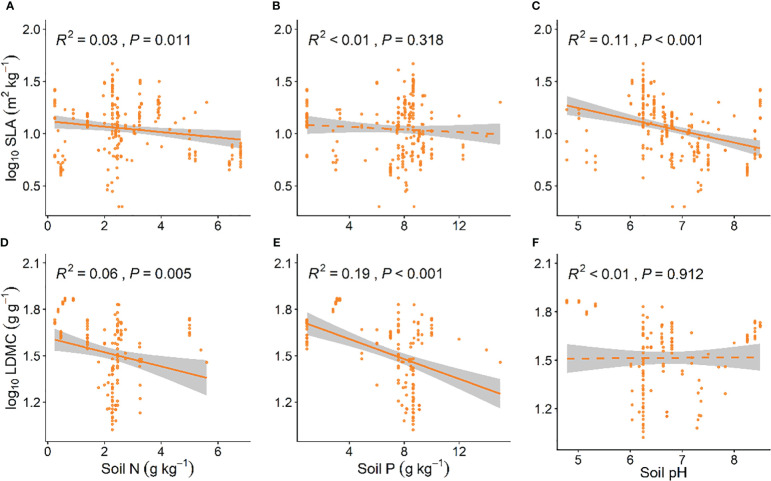
Linear relationships between soil nutrient factors and SLA **(A–C)** and LDMC **(D–F)**. Both SLA and LDMC data have been log-transformed. Soil factors include: soil nitrogen (N) content, available soil phosphorus (P) content, and soil pH. *R* ^2^ indicates the model’s goodness of fit, and the *P*-value indicates the level of significance. The shaded area shows a 95% confidence interval.

Grassland plant SLA is significantly negatively correlated with soil N and soil pH (*P* < 0.05, **Figure 4A**; *P* < 0.001, **Figure 4C**), and LDMC decreases with an increase in soil N and soil P (*P* < 0.01, **Figures 4D, E**). As soil nutrients increase, the resource utilization strategy of grassland plants shifts from a “conservative” mode to a “faster investment-return” survival strategy (**Figures 4B, F**).

### The dominant environmental factors affecting investment strategies for grassland plants

**Figure 5:**
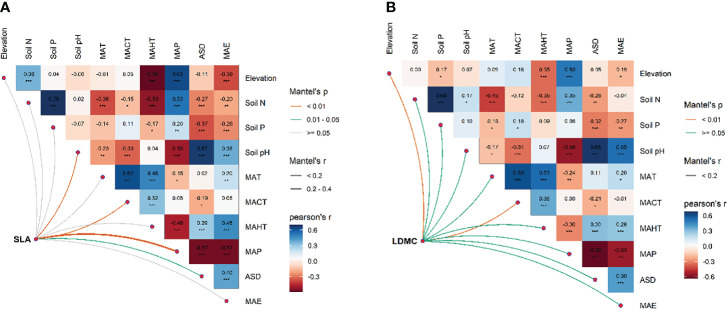
Multivariate correlation analysis among potential influencing factors for SLA **(A)** and LDMC **(B)**. Both SLA and LDMC data have been log-transformed. Influencing factors include: climatic factors [mean annual temperature (MAT), coldest month mean temperature (MACT), hottest month mean temperature (MAHT), mean annual precipitation (MAP), mean annual evaporation (MAE), and annual sunshine duration (ASD)] and soil nutrient factors [soil nitrogen content (Soil N), available soil phosphorus content (soil P), and soil pH)]. Asterisks indicate levels of significance (***P < 0.001; **P < 0.01; *P < 0.05).

**Figure 6:**
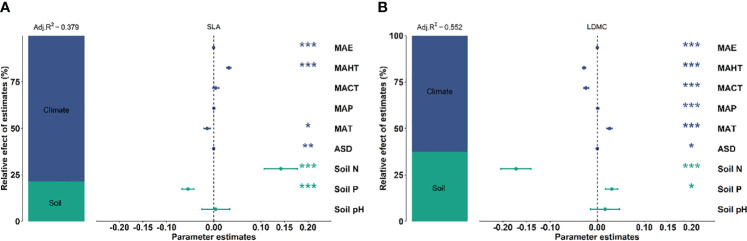
Relative impacts of climatic and soil nutrient factors on SLA **(A)** and LDMC **(B)** based on variance decomposition models. Both SLA and LDMC data have been log-transformed. Climatic factors include: mean annual temperature (MAT), coldest month mean temperature (MACT), hottest month mean temperature (MAHT), mean annual precipitation (MAP), mean annual evaporation (MAE), and annual sunshine duration (ASD). Soil nutrient factors include: soil nitrogen content (Soil N), available soil phosphorus content (Soil P), and soil pH. The relative importance of each factor is represented as the percentage of explained variance (left panel). The mean parameter estimates of the model predictors (right panel) are presented as standardized regression coefficients ± 95% confidence intervals and *P*-values for each predictor are given as: ***P < 0.001; **P < 0.01; *P < 0.05.

**Figure 7:**
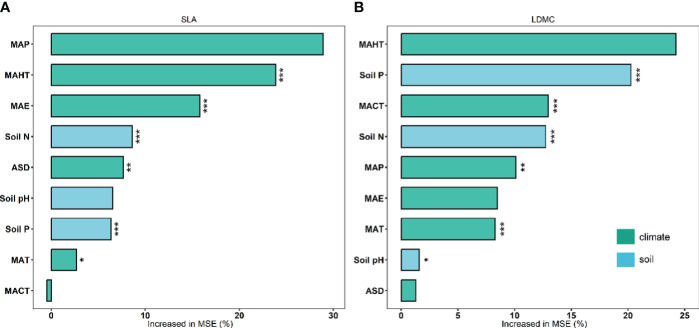
Independent contributions of each climatic and soil nutrient factor to SLA **(A)** and LDMC **(B)**. Both SLA and LDMC data have been log-transformed. Climatic factors include: mean annual temperature (MAT), coldest month mean temperature (MACT), hottest month mean temperature (MAHT), mean annual precipitation (MAP), mean annual evaporation (MAE), and annual sunshine duration (ASD). Soil nutrient factors include: soil nitrogen content (Soil N), available soil phosphorus content (Soil P), and soil pH. Percentage increase in mean square error (MSE, %) of variables was used to estimate the importance of these predictors, and higher MSE% values implied more important predictors. Asterisks indicate levels of significance (***P < 0.001; **P < 0.01; *P < 0.05).

There exists significant correlation between the potential influencing factors (**Figure 5**). Variance decomposition results show that climatic factors contribute more to SLA and LDMC than soil factors (**Figure 6**), dominating the resource utilization strategy of grassland plants. MAP and MAHT make the largest independent contributions to the spatial variability of SLA and LDMC in grasslands (**Figure 7**).

### The direct and indirect effects of elevation on investment strategies for grassland plants

**Figure 8:**
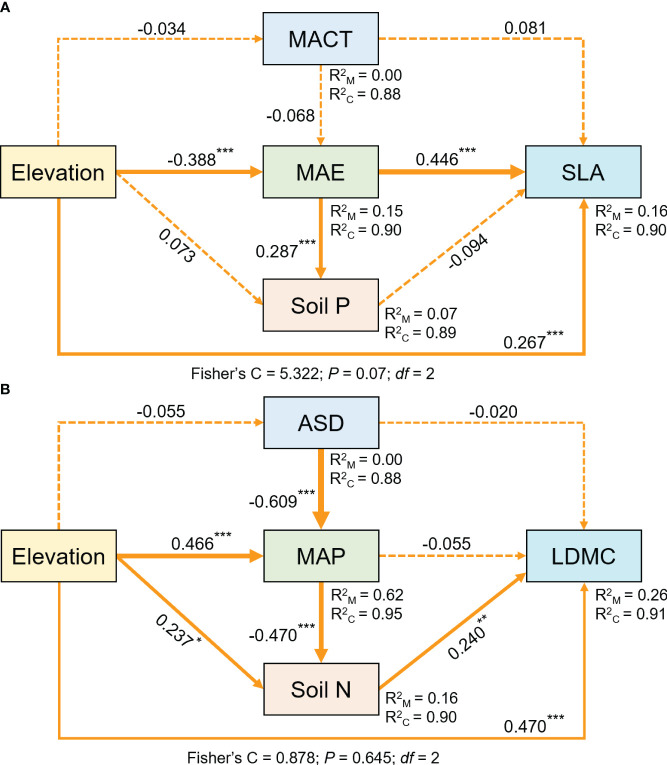
The structural equation model shows the direct and indirect effects of elevation on SLA **(A)** and LDMC **(B)**. Both SLA and LDMC data have been log-transformed. Arrows represent hypothesized impact pathways, with numbers next to the arrows indicating standardized path coefficients, and asterisks denoting levels of significance (***P < 0.001; **P < 0.01; *P < 0.05). The thickness of the arrows represents the relative magnitude of the path coefficients. R^2^_M_ and R^2^_C_ represent marginal and conditional *R* ^2^, respectively.

Structural equation model results indicate that elevation not only directly affects SLA and LDMC, but also influences them through soil nutrient factors and climatic factors, with its direct effects being greater than its indirect effects (**Figure 8**).

## Discussion

### Investment strategies for grassland plants at different elevations

Environmental conditions along elevation gradients affect the adaptability, growth, survival, and functionality of plants, which adopt different resource utilization strategies under varying environmental conditions (Islam et al., 2024). Our research findings indicate that herbaceous plants in lower elevation areas have higher SLA and lower LDMC, while those at higher elevations exhibit lower SLA and higher LDMC (**Figures 1B, C**). This is primarily due to changes in climatic and soil nutrient conditions caused by different elevation gradients (Sundqvist et al., 2013; Gong et al., 2020). In lower elevation areas, where the climate is milder and the water and thermal conditions are more favorable, plants generally adopt a “faster investment-return” resource utilization strategy. They increase SLA and decrease LDMC, achieving rapid growth and development at lower construction costs, which allows them to gain more resources and competitive advantages for a wider ecological niche (Kunstler et al., 2016; Niu et al., 2018). Conversely, in higher elevation areas, where temperatures are low, ultraviolet radiation is strong, the growing season is short, and soil nutrient availability is poor, the living conditions are harsher. Therefore, plants usually adopt a “slower investment-return” strategy, increasing LDMC and decreasing SLA to prolong leaf lifespan, and they invest more in leaf thickness construction to combat adverse conditions and enhance survival rates (Gong and Gao, 2019).

Our study findings indicate that grassland plants adopt different resource utilization strategies across different elevations, shifting from a “fast investment-return” acquisitive strategy at lower elevations to a “slow investment-return” conservative strategy at higher elevations, which is consistent with the study findings of Wieczynski et al. (2019); Kramp et al. (2022), and Feng et al. (2023). In the context of global climate change, understanding how these strategies affect grassland resilience and productivity is crucial. Conservative strategies at higher elevations may enhance resilience by allocating resources efficiently under harsh conditions, potentially buffering against climate fluctuations (Wang et al., 2022a). Meanwhile, acquisitive strategies at lower elevations might maximize productivity but could also increase vulnerability to environmental stressors (Zemunik et al., 2015). Balancing these strategies through adaptive management could optimize grassland health and sustainability amid changing climates (Wang et al., 2022b). Through scientifically sound management measures, it is possible to effectively enhance grassland resilience and productivity, protect biodiversity, enhance plant adaptation to climate change, and maintain the ecological functions of grasslands (Moore and Schindler, 2022).

### The impact of climate factors on investment strategies for grassland plants

Our data indicates that with increasing temperature, SLA generally increases while LDMC significantly decreases (**Figures 3B, C**), which is contrary to the findings of Gong and Gao (2019) and Wang et al. (2022a). This may be because as temperatures increases, plants typically increase leaf area to enhance photosynthesis, thus more efficiently utilizing available light. A higher SLA indicates thinner leaves, which facilitates light capture and gas exchange in photosynthesis (Huang et al., 2020). An increase in leaf area with significantly thinner leaves is related to a reduction in leaf tissue thickness caused by a decrease in the number of cell layers and cell size in the epidermal, palisade, and spongy tissues (Hartikainen et al., 2009; Jin et al., 2011). In cold environments, cell growth is restricted, leading to smaller cell sizes, increased intracellular contents, and more cell layers. These changes increase the content of proteins and secondary metabolites in leaves, slowing down freezing rates and reducing frost damage and stress, thus enhancing the plant’s cold resistance. This may explain the increase in LDMC and the decrease in SLA (Tardieu and Granier, 2000; Atkin et al., 2006; Usadel et al., 2008). In addition, our results also indicate that with increasing rainfall, SLA tends to increase (**Figure 2D**), which is consistent with the study findings of Akram et al. (2023). Previous studies have shown that under low precipitation, plants experience water stress and reduce stomatal opening to minimize water loss, increasing LDMC and reducing SLA, and invest more in leaf construction, increasing the thickness of epidermal cells, particularly palisade tissues, making cells more compact and reducing water loss from the plant (Galmés et al., 2013). Nevertheless, as precipitation increases, soil conductivity and photosynthetically active radiation decrease, weakening grassland plant photosynthesis. Plants increase SLA and decrease LDMC to capture more light, enhancing photosynthesis and acquiring more resources to sustain life activities and growth (Kröber et al., 2015). Furthermore, with an increase in ASD, SLA shows a decreasing trend (**Figure 2F**). This may be because when sunlight duration is insufficient, plants reduce leaf thickness and density, increasing SLA to expand the leaf area for light capture. This improves photosynthetic efficiency under low light conditions, thereby enabling plants to survive periods of insufficient light (Lusk et al., 2008; Coble and Cavaleri, 2015). In addition, our results also indicate that with increasing evapotranspiration, SLA shows a decreasing trend (**Figure 2E**). Research has shown that evapotranspiration is often related to soil moisture content. Specifically, in dry soil areas, evapotranspiration tends to significantly increase, typically resulting in lower soil moisture content (Liu et al., 2023). Therefore, when evapotranspiration is high and soil moisture is severely deficient, plants experience water stress and respond by increasing LDMC and reducing SLA to minimize water loss.

### The impact of soil factors on investment strategies for grassland plants

Most of the nutrients required for plant growth are provided by the soil, and soil nutrients are closely related to plant resource utilization strategies (Gao et al., 2019; Joswig et al., 2022). Our study results show that soil nitrogen and phosphorus content are negatively correlated with LDMC (**Figures 4D, F**), which is consistent with the study findings of Hodgson et al. (2011). The level of soil nitrogen often indicates the overall nutrient status of the soil, with higher soil nitrogen content associated with more fertile soil. In contrast, in conditions of low soil nitrogen, where nutrients are scarce, nutrient conservation becomes crucial for plants, which then increase LDMC and grow slowly, adopting a “slower investment-return” resource utilization strategy (Ordoñez et al., 2009). Additionally, soil phosphorus content significantly influences the composition of microbial communities (Finkel et al., 2019). An increase in soil phosphorus enhances microbial activity and soil nutrient availability, prompting plants to increase SLA and grow rapidly and develop. Furthermore, our results indicate SLA significantly decreases with increasing soil pH (**Figure 4C**). Tao et al. (2019) found that under acidic conditions, plants exhibit higher SLA and lower LDMC, rapidly acquiring nutrients to achieve maximum growth, adopting a “faster investment-return” resource utilization strategy, our results are similar to them.

### The impact and relationship of elevation and environmental factors on grassland plant investment strategies

Elevation not only directly affects plant resource utilization strategies, but also indirectly influences them by modulating climatic and soil nutrient factors, with its direct effects being greater than its indirect effects (**Figure 8**). As the elevation gradient increases, key environmental factors affecting plant growth, including climatic factors and associated soil factors, undergo significant changes (Sundqvist et al., 2013). Our study results also confirm that climate and soil factors vary with elevation. In lower elevation areas, the climate is suitable and hydrothermal conditions are favorable for plant survival. In contrast, in higher elevation areas, harsh environmental conditions such as low temperatures, intense ultraviolet radiation, and low oxygen environments have a direct and significant impact on plant physiological processes, forcing plants to adapt directly in order to survive (Abbas et al., 2022). Moreover, the environmental changes caused by elevation changes (e.g., atmospheric pressure, radiation, and duration of sunlight) are rapid and direct. On one hand, these environmental factors directly act on plants, affecting their growth and development processes, leading to changes in their resource utilization strategies and rapid adjustments in key plant functional traits. On the other hand, elevation indirectly affects plant resource utilization strategies by influencing climatic and soil factors. This is usually more complex and involves a time lag.

Research has found that differences in SLA and LDMC among grassland plants are primarily driven by hydrothermal conditions (Wang et al., 2022a). For example, precipitation and temperature can not only directly affect the physiological and biochemical characteristics of plants, but also influence plant resource utilization strategies by affecting microbial activity, accelerating the leaching and transformation of soil nutrients (Yang et al., 2022; Zhu et al., 2023). The availability of soil nutrients depends on climatic factors and soil microbes (Fang et al., 2021; Gao et al., 2021). Therefore, soil nutrients and climatic factors together influence the resource utilization strategies of herbaceous plants, with the contribution of climatic factors being greater than that of soil nutrient factors.

This study investigated the resource utilization strategies of grassland plants across different elevation gradients and their driving factors. The findings bear significant ecological implications for grassland management and conservation under global climate change. However, the study has limitations as the elevation data were sourced from multiple grasslands in China rather than from different elevations within the same region, leading to considerable habitat heterogeneity. High habitat heterogeneity implies diverse environmental conditions within the same elevation range, potentially compromising the comprehensive representation of unique ecological conditions at each elevation. Moreover, in highly heterogeneous habitats, interactions among different plant species and between plants and their environment may be more complex, making it challenging to understand and elucidate the relationships between plant resource utilization strategies and elevation gradients, as well as their driving factors. Therefore, future research addressing this scientific question at a larger scale needs to carefully consider the influence of habitat heterogeneity. This can be achieved by collecting long-term monitoring data, integrating more research datasets, and employing advanced statistical models to more accurately predict plant resource utilization strategies and their responses to climate change.

## Conclusions

Our study examined the differences in resource utilization strategies of grassland plants across various elevation gradients in China and their influencing factors. The results indicate that grassland plants exhibit different resource utilization strategies at different elevations. With increasing elevation, SLA significantly decreases while LDMC significantly increases, shifting from a “faster investment-return” strategy in lower elevations to a “slower investment-return” strategy in higher elevations. These changes are primarily regulated by climatic factors, among which MAP and MAHT are the relatively independent key climate factors with the greatest contribution. Soil nutrient factors also play a non-negligible coordinating role. This study highlights the substantial impact of elevation on grassland plant resource utilization strategies, which is crucial for understanding the elevational patterns of grassland plant resource utilization strategies under global change. The different resource utilization strategies of grassland plants at high and low elevations have important implications for grassland management and conservation. Building on our findings, future research could focus on temporal changes in grassland plant resource utilization strategies to better understand these strategies under the background of global climate change.

## Data availability statement

The raw data supporting the conclusions of this article will be made available by the authors, without undue reservation.

## Author contributions

JY: Writing – original draft, Writing – review & editing. YJ: Formal analysis, Funding acquisition, Resources, Writing – original draft, Writing – review & editing. JW: Resources, Software, Validation, Writing – original draft, Writing – review & editing. XM: Funding acquisition, Project administration, Writing – review & editing. JG: Conceptualization, Data curation, Formal analysis, Funding acquisition, Investigation, Methodology, Project administration, Resources, Software, Supervision, Validation, Visualization, Writing – original draft, Writing – review & editing.

## Supplementary material

Supplementary Figure 1 Linear relationships between climatic factors and elevation. (**A**) mean annual temperature (MAT); (**B**) coldest month mean temperature (MACT); (**C**) hottest month mean temperature (MAHT); (**D**) mean annual precipitation (MAP); (**E**) mean annual evaporation (MAE); (**F**) annual sunshine duration (ASD). LDMC data have been log-transformed. *R* ^2^ indicates the model’s goodness of fit, and the *P*-value indicates the level of significance. The shaded area shows a 95% confidence interval.

Supplementary Figure 2 Linear relationships between soil nutrient factors and elevation. Soil nutrient factors include: soil nitrogen (N) content (**A**), available soil phosphorus (P) content (**B**), and soil pH (**C**). *R* ^2^ indicates the model’s goodness of fit, and the *P*-value indicates the level of significance. The shaded area shows a 95% confidence interval.
