# Supplementary figures and images for: Climate factors dominate the elevational variation in grassland plant resource utilization strategies
Source: Front Plant Sci. 2024 Aug 7;15:1430027. doi: 10.3389/fpls.2024.1430027 (PMC11335560; doi:10.3389/fpls.2024.1430027)

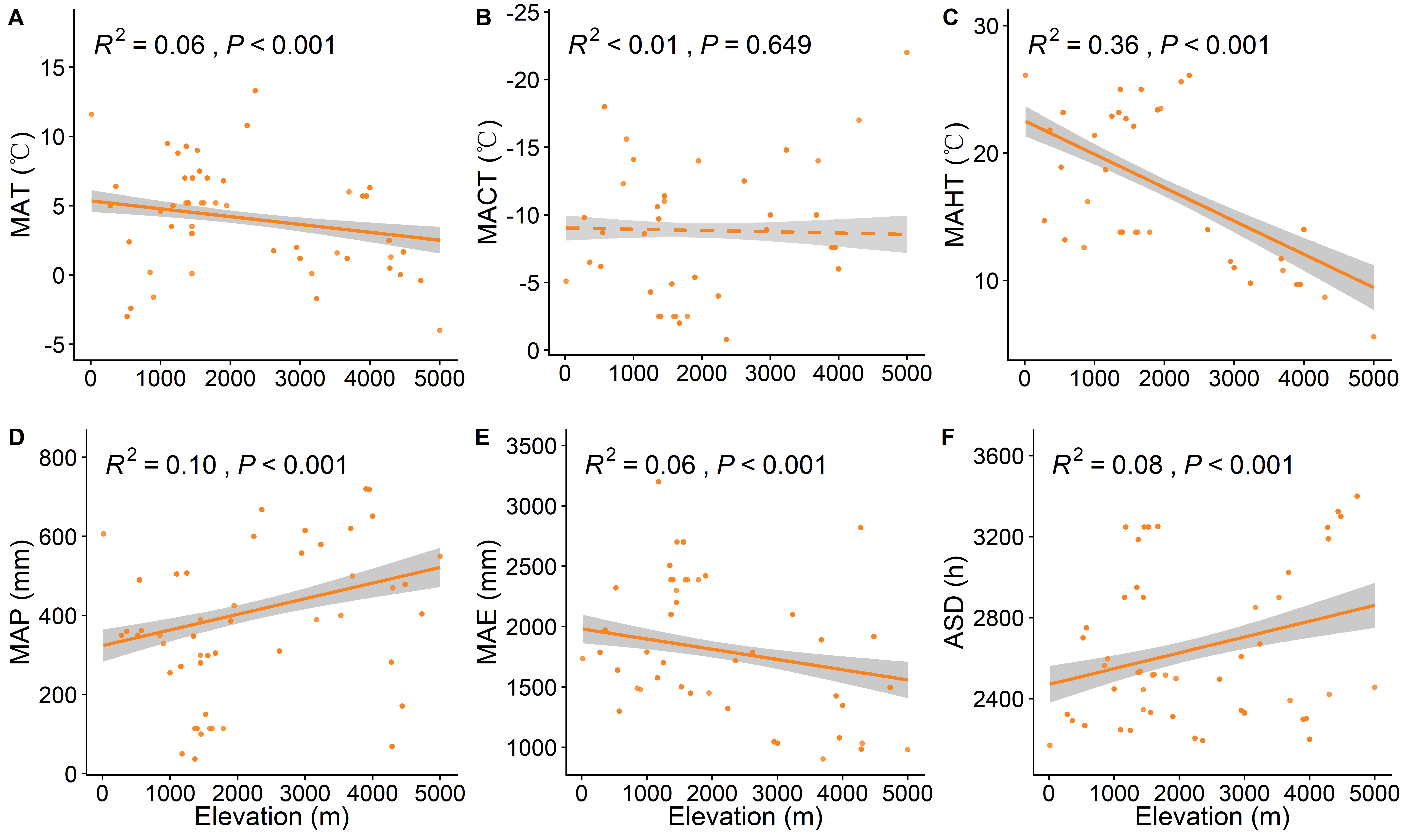

Supplement: Supplementary Figure 1 — Linear relationships between climatic factors and elevation. (A) mean annual temperature (MAT); (B) coldest month mean temperature (MACT); (C) hottest month mean temperature (MAHT); (D) mean annual precipitation (MAP); (E) mean annual evaporation (MAE); (F) annual sunshine duration (ASD). LDMC data have been log-transformed. R 2 indicates the model’s goodness of fit, and the P-value indicates the level of significance. The shaded area shows a 95% confidence interval. [file Figure_1.tif]

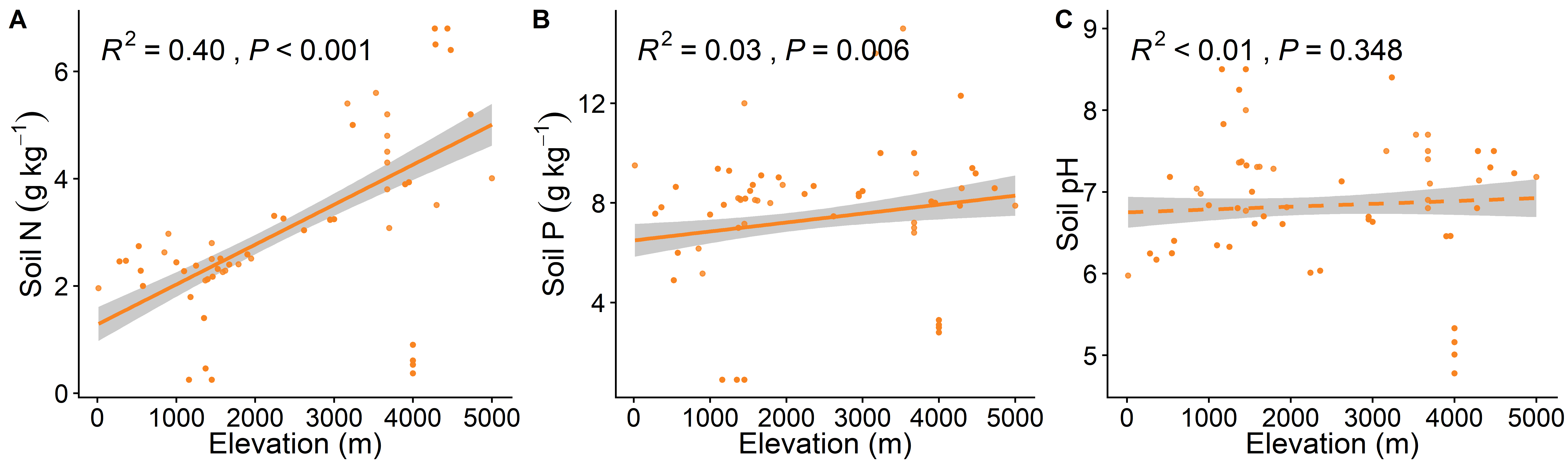

Supplement: Supplementary Figure 2 — Linear relationships between soil nutrient factors and elevation. Soil nutrient factors include: soil nitrogen (N) content (A), available soil phosphorus (P) content (B), and soil pH (C). R 2 indicates the model’s goodness of fit, and the P-value indicates the level of significance. The shaded area shows a 95% confidence interval. [file Figure_2.tif]
